# Behaviour during transportation predicts stress response and lower airway contamination in horses

**DOI:** 10.1371/journal.pone.0194272

**Published:** 2018-03-22

**Authors:** Barbara Padalino, Sharanne L. Raidal, Peter Knight, Pietro Celi, Leo Jeffcott, Gary Muscatello

**Affiliations:** 1 Faculty of Veterinary Science, School of Life and Environmental Sciences, The University of Sydney, Sydney, NSW, Australia; 2 Department of Veterinary Medicine, University of Bari, Bari, Italy; 3 College of Veterinary Medicine, City University of Hong Kong, Kowloon, Hong Kong, HKSAR; 4 School of Animal and Veterinary Sciences, Charles Stuart University, Wagga Wagga, NSW, Australia; 5 Discipline of Biomedical Science, School of Medical Sciences, Sydney Medical School, The University of Sydney, Lidcombe, NSW, Australia; 6 DSM Nutritional Products, Animal Nutrition and Health, Columbia, Maryland, United States of America; 7 Faculty of Veterinary and Agricultural Sciences, The University of Melbourne, Parkville, VIC, Australia; Cardiff University, UNITED KINGDOM

## Abstract

This study aimed to document the effects of an eight hour journey on behavioural, clinical, haematological, environmental and respiratory parameters, and to identify possible associations between factors. Twelve horses underwent clinical examination, respiratory endoscopy with tracheal wash (TW) aspiration, and collection of venous and arterial blood before (BJ) and after the journey (AJ). TW were submitted for conventional quantitative bacteriological evaluation and genetic microbiome analyses. Behaviour was assessed in stables prior to transportation and throughout the journey. Transportation caused mild, but significant, effects on fluid and electrolyte balance and an acute phase response, characterized by neutrophilia, hyperfibrinogenaemia and hyperglobulinaemia. The proportion of neutrophils in TW, tracheal mucus and TW bacterial concentration was increased AJ, with preferential replication of *Pasteurellaceae*. Horse behaviour *en route* predicted clinical and respiratory outcomes. The frequency of stress related behaviours was greatest in the first hour of the journey, and balance-related behaviours were most common in the final hour of the journey. Horses which lowered their heads less frequently *en route* and showed more stress-related behaviours had higher physiological stress (serum cortisol and heart rate on arrival), increased tracheal mucus and inflammation scores, and higher TW bacterial concentration AJ (P<0.05). Six horses with abnormal lung auscultation AJ proved to have had higher tracheal inflammation scores at preloading (P = 0.017), an overall higher concentration of bacteria in their TW (P = 0.013), and an increased percentage of neutrophils in TW at five days AJ (P = 0.003) in comparison to the other horses. While transport-related health problems are multifactorial, clinical examination, including auscultation and endoscopic inspection of the lower respiratory tract before and after journey, and behavioural observation *en route* may identify animals at increased risk of transport associated respiratory disease.

## Introduction

Respiratory disease is a common consequence of equine transportation [1–6]. The risk of transport associated respiratory disease in the horse may be influenced by pre-existing inflammation of the airways [7, 8], journey duration [9–11], head position [2, 12, 13], air quality inside the truck (increased concentration of ammonia and carbon monoxide gas [14, 15]), transport-associated effects on immunity [16–19], thermal stress *en route* [20] and transport-related dehydration [12, 21].

Transport-associated pneumonia in horses is not linked to a specific pathogen, rather a mixture of different bacterial species have been linked to transport related lung infections with varying involvement of the pleural space [22]. It remains unclear why some horses develop illness and others do not. Given that multiple factors have been linked to the development of respiratory disease following transport, there is need for a multidisciplinary approach when studying equine transport, such that predictors of airway contamination can be explored with respect to the horse and its environment. The current study was designed, therefore, to determine the effects of an eight hour truck journey on various behavioural, clinical, environmental and respiratory parameters in horses, and to identify possible associations between these factors. It is hypothesised that behavioural responses could be related to the mental and physiological ‘stress’ of transportation, as determined by plasma cortisol concentrations, and that behavioural responses could drive anatomical position of the head during transport and thereby influence tracheal mucus score and bacterial contamination of the lower respiratory tract.

## Material and methods

### Animals

Twelve horses (6 Standardbreds and 6 Thoroughbreds; 7 geldings and 5 mares), aged from 3 to 8 years (mean 4.9 ± 1.9 years), with mean body condition score (BCS) of 2.2 ± 0.4 [23], were recruited into this study. The horses were randomly selected from the research herd of Charles Sturt University, were healthy on veterinary evaluation and judged fit for transportation [24]. All were well accustomed to handling and had previous experience of transportation. The protocol was approved by the Charles Stuart University Animal Care and Ethics Committee (Project Number 14/037). Based on previous results for bacterial contamination of the lower respiratory tract [1] and cortisol concentration [25] associated with transportation, power analysis demonstrated that results from 12 horses would permit recognition of a doubling of bacterial load and increases of plasma cortisol of >50 nmol / L with >90% power and α = 0.05.

### Experimental protocol

On day 1, all animals were clinically assessed, weighed, and dewormed by two research team members (BP and SR). All horses were normal on routine haematology and serum biochemistry on recruitment to the study. The animals then underwent two weeks of acclimation. During the first week, the horses were kept on pasture. During the second week, the horses were kept in single boxes (4x4 m), with shavings as bedding. Except during transportation, they were stabled for the rest of the experiment, spending at least one hour a day in a yard. They were fed lucerne hay and oats twice a day (08.00; 18.00 h), and had water *ad libitum*. The diet was calculated individually to meet maintenance requirements.

The horses were transported in consignments of six over two identical trips for 8 hours on different days, 48 hours apart. The horses travelled in the same 6-horse commercial truck (Freighter, Fuso, Mitsubishi, Japan), with venturi vents and louvres. The horses travelled in individual stalls (0.80 m width x 2.30 m length), in a sideways position, restrained by rubber cords. The cord was attached to the low ring of the head collar, so animals were able to turn their head and lower their head below wither height, such that they could touch their carpus, but not the floor, with their nose. The truck was driven by the same experienced driver for both trips. To comply with the occupational health and safety requirements, a rest stop for the driver, from 12.35 to 13.00, was included. During the rest stop, the horses were not unloaded and the truck was parked in the shade. Horses were not offered food or water at any time during transport.

Horse behaviour and environmental parameters were recorded when the horses were at rest in their stables (the day before the journey), and *en route*. Air samples were collected in the stable (the day before and one day after the journey) and in the truck at preloading and unloading on both journeys. During the trial all experimental procedures were sequenced according to invasiveness and possible impact on animal welfare. Experimental procedures were conducted in the following order at each sampling point: clinical examination, venous blood collection, arterial blood collection and upper respiratory endoscopic examination. All sampling protocols are summarized in S1 Table.

### Clinical examination

Clinical examination was performed by an experienced equine veterinarian (BP) prior to departure (preloading), at unloading, 12 hours after journey (AJ), and 1, 2, 3, 4, 5 days AJ, as previously reported [19]. Briefly, horses’ demeanour, heart rate (HR), respiratory rate (RR), rectal temperature (RT), mucous membrane characteristics (colour, moisture and capillary refill time), pulmonary and gastrointestinal auscultation were documented at each time, according to established clinical practices. Palpable lymph nodes, presence of nasal discharge and coughing were also recorded. All clinical examinations were performed without knowledge of endoscopic or laboratory findings for each horse. The body weight (BW) of all horses were recorded at pre-loading, unloading, 1 and 5 days AJ using a digital scale (Ibeef®,Farm, Weigh System, Ruddweigh^TM^ 200, Tauranga, New Zealand).

### Environmental parameters and air quality

A weather tracker (Kestrel: 4000 Pocket Weather Tracker, Nielsen-Kellerman, USA) and a gas detector (Dräger X-am 5000, Dräger Safety AG & Co., Lübeck, Germany) were placed inside a box at horse head height whilst horses were stabled on the day before the journey, and were placed in the last bay of the truck at horse head height during the two trips. The weather tracker recorded the following climate parameters: temperature (T, °C), humidity (H, %), heat-index (HI, °C), wind speed (W, Km/h) every 5 minutes during the journey and the concentration of oxygen (O_2_, vol%), ammonia (NH_3_, ppm), hydrogen sulphide (H_2_S, ppm), carbon monoxide (CO, ppm), methane (CH_4_, % of lower explosive limit (LEL)) was monitored continuously by the gas detector. Air samples were collected using an air sampler (Coriolis μ, Bertin Instrument, CNIM Group, Montigny-le-Bretonneux, France) to assess the concentration of bacteria in the air inside the truck and the stable. Air samples were collected in the middle of the truck before loading and immediately after unloading, with the ramp opened. Air samples were collected in the middle of an unoccupied box the day before and the day after the journey, with the door of the box opened. Air was aspirated at 300L/min for three minutes into a sterile sampling cone with 0.1% peptone water. The samples were put in a sterile container and kept on ice until analysis within 4 hours of collection. The air samples underwent the same bacteriological evaluation as described for the tracheal wash samples below (section 8.1 and 8.2).

### Behavioural parameters

Horses were recorded in their stables by a security camera system (TechView DVR Kit, Model Number QV-3034) placed in each box. Behaviour was recorded in the boxes for 1 hour in the middle of the day (from 12.00 to 13.00) on the day prior to travel, while horses were denied access to water and feed. During transport, a camera was placed in each single box of the trailer, pointing toward the horse’s head, enabling the horse’s behaviour to be recorded continuously during the journey, including the rest stop. A behaviour sampling ethogram (Table 1) was developed based on those used previously to study behaviour during transportation [26–28]. Videos were analysed by an experienced ethologist (BP) using a time window defined as the first 25 minutes of each hour of the journey, during the rest stop, and the last 25 minutes of recording whilst stabled. This time window was selected to permit direct comparison with behaviours observed during the rest stop. Behavioural assessment was performed independently of clinical and laboratory assessment for each horse.

**Table 1:** Behaviour sampling ethogram.

Behaviour	Description	
**Behavioural events related to stress** (Expressed as frequency) (n/25 min)		
**Evasive behaviour/pulling back**	The horse tries to escape from the truck, it pulls back trying to break the rope	
**Explorative behaviour/sniffing**	The horse sniffs around, it sniffs some area of the truck/box	
**Licking/chewing**	Opening of mouth with extension and retraction of tongue, lip smacking without tongue extension, lateral jaw movements involving partial opening of the lips [29]	
**Licking the truck/wall**	The horse licks part of the truck/ box (wall, stall rails)	
**Nose outside**	The horse puts his nose between the bars of the truck/box	
**Pawing**	One front leg is lifted from the ground slightly, then extended quickly in a forward direction, followed by a movement backward, dragging the toe against the floor in a digging motion [30]	
**Touching rubber tie cord**	The horse touches the rubber cord which he is tied with	
**Turning the head**	The horse turns his head and neck to the right or to the left appearing to look at his flank	
**Total stress related behaviours**	Sum of the behavioural events related to stress	
**Behavioural Events related to balance** (Expressed as frequency) (n/25 min)		
**Backward movements**	The horse steps backward	
**Forward movement**	The horse steps forward	
**Lateral movements**	The horse steps sideways	
**Leaning on stall rails**	The horse gently leans laterally against one of the two stall rails	
**Loss of balance/dashing on the partitions**	The horse losses his balance and crashes/bumps on one stall rails	
**Total balance related behaviours**	Sum of the behavioural events related to balance	
**Other behavioural Events** (Expressed as frequency) (n/25 min)		
**Head tossing/shaking**	The horse shakes its head suddenly, violently and frequently	
**Interaction with neighbours**	The horse interacts with one of his neighbour trough the stall rails, they sniff each other	
**Biting neighbour**	The horse bites the neighbour	
**Look outside**	The horse looks outside, head and ears pointing outside	
**Lowering the head**	The horse lows his head below the withers height	
**Shaking head**	The horse shakes its head	
**Yawning**	An involuntary sequence consisting of mouth opening, deep inspiration, brief apnoea, and slow expiration [31]	
**Total behavioural events**	Sum of all behavioural events	
**Head down duration** (s/25 min)	The horse stays his head at the level or below withers height	

The frequency and duration of each identified behaviour was assessed for each horse during 25 minute time windows for stabled horses, each hour during transportation and during the rest stop.

### Venous and arterial blood samples

Venous blood was collected by jugular venepuncture at pre-loading, unloading, 12 and 24 hours AJ, and 5 days AJ. Blood samples were collected into heparinised, plain and EDTA-Vacutainer tubes (Becton Dickinson, Franklin Lakes, NJ). After collection, blood samples were kept at 4^°^C and analysed within 4 hours of collection.

Routine haematology and serum biochemistry parameters were determined by a commercial laboratory (Veterinary Diagnostic Laboratory, Charles Sturt University, Wagga, AU) using standard laboratory processes and equipment (Cell Dyn 3700 Cell Counter; Abbott, Chicago, IL and Konelab 20XT photometer; Thermo Fisher Scientific, Finland, EU). Fibrinogen was calculated by heat precipitation [32]. Serum amyloid A was determined by commercial kit (Equinostic, DN, Equibnostic, Copenhagen, Denmark) using a dedicated spectrophotometer (EVA, Equinostic, Copenhagen, Denmark) [33]. Cortisol concentration was assessed in serum samples by radioimmunoassay (RIA) as previously described [19].

Arterial blood samples were collected anaerobically from the transverse facial artery with a 3 mL heparinized syringe at preloading, unloading and 24 hours AJ. Samples were kept at 4^°^C and analysed within 2 hours of collection using a blood gas analyser (Gem Premier 3500; Diamond Diagnostic, Holliston, MA, USA).

### Respiratory endoscopy and tracheal wash (TW) collection

Respiratory endoscopy was performed using a 1.35m, 9.2mm outer diameter, fibre-optic video endoscope (Olympus, Gif Q165, distributed by Ausvet Endoscopy, Melbourne, Australia) connected to a video processor (Olympus CV-160 Exerm, Ausvet Endoscopy, Melbourne, Australia) two days prior to the journey, at unloading, 24 hours AJ and 5 days AJ. All horses were sedated using xylazine and acepromazine (0.4 mg/kg BW and 0.02 mg/kg BW, respectively) prior to the procedure. Airway inflammation and tracheal secretions were subjectively graded (S2 Table) [34] and TW was obtained trans-endoscopically by lavage and aspiration of 30 mL of room temperature 0.9% saline via a guarded polyethylene catheter (EMAC800, Mila International, Ausvet Endoscopy, Melbourne, Australia) passed through the endoscope instrument channel. Gross characteristics of TW samples were recorded using a clinical sheet (S2 Table). The endoscope and the instrument channel were disinfected with chlorhexidine scrub and 70% alcohol between each horse examination.

#### Tracheal wash (TW) cytology

Two smears were prepared directly from each TW sample or using a cytospin centrifuge (Shandon, Cytospin III, GMI, Minnesota, USA) for dilute samples. The smears were stained using Wright-Giemsa stain (Hema-tek stain pack, Sigma-Aldrich Pty Ltd, Castle Hill, NSW, Australia). Four differential cell counts (DCC) each of 100 cells were made using high-power (100X) light microscopy. Differential cell counts of all samples were performed (by BP), blinded to horse identification, and expressed as a percentage.

### Bacteriology

#### Quantitative and phenotypic bacteriology

One millilitre of each air sample and TW aspirate was 10-fold serially diluted with sterile saline. A 100 μL aliquot of each dilution was inoculated onto 5% sheep blood agar (SBA) plates and incubated under aerobic conditions at 37^°^C and 6% CO_2_. Colonies were counted on plates with fewer than 300 colonies after 48h incubation and expressed as colony-forming units (CFU) per mL (converted to log_10_ CFU/mL for analysis). Bacterial isolates were identified according to phenotypic definitions (S3 Table) based on colony morphology (shape, size, elevation, surface, edge, colour, opacity, consistency, odour), presence of haemolysis (nil, complete or incomplete haemolysis), Gram staining and basic bacterial cellular morphology (i.e rods, cooci etc.) and biochemical characteristics primarily the presence or absence of catalase enzyme for Gram positive bacteria (i.e. catalase test) or the presence or absence of cytochrome oxidase from Gram negative bacteria (i.e oxidase test).

#### Bacterial microbiome analysis

Bacterial DNA was extracted from 1 mL of TW with the addition of 0.5 mL of dithiothreitol (150μg/mL) using a commercial kit (Sputum DNA isolation kit, NorgenBiotek Corp, Thorold, ON, Canada). Bacterial DNA was extracted from air samples using a commercial kit (Mobio Soil Powerlyzer kit; Qiagen, Carlsband, Ca, USA). Extracted DNA samples were evaluated for quantity and quality using a NanoDrop-2000 (Thermo Fisher Scientific Inc., Scoresby, Victoria, Australia). Purified DNA samples were sent to the Australian Genome Research Facility (AGRF) for microbial diversity profiling based on sequencing of partial 16s rRNA gene amplicons (V1 forward primer (27F): AGAGTTTGATCMTGGCTCAG; V3 reverse primer (519R): GWATTACCGCGGCKGCTG). Amplicons from the primary PCR were indexed by secondary PCR and resultant amplicons were quantified by fluorometry and normalised. The equimolar pool was then measured by qPCR and sequenced (Illumina MiSeq; San Diego, CA, USA) with 2 x 300 base pairs paired-end chemistry. Paired-end reads were assembled by aligning forward and reverse reads using PEAR (version 0.9.5) [35]. Primers were trimmed using Seqtk (version 1.0) [36] and trimmed sequences were processed using Quantitative Insights into Microbial Ecology (QIIME 1.8) [37] USEARCH (version 8.0.1623) [38, 39] and UPARSE [39] software. Sequences were clustered using “rdp_gold” database [40] as the reference and each read was mapped back to an operational taxonomic unit (OTU) with a minimum identity of 97% using Qiime taxonomy and Greengenes database (version 13_8, Aug 2013) [41]. The number of reads in each OTU was recorded for all samples.

### Statistical analysis

All data were explored initially using summary statistics and normal distribution of all quantitative data was checked using the Anderson-Darling test. Gen Stat Version 14 (VSNi International) was used for χ^2^ analyses and linear regression, and ordinal logistic analysis was performed using R [42]. All other analyses were performed using SAS (SAS, version 9, 1999). For all statistical analyses, a P value of <0.05 was considered significant.

#### Environmental and air quality parameters inside the truck and the boxes

Environmental data were analysed using ANOVA to determine the effect of situation (box 1, box 2, trip 1, trip 2) on the following variables: T, H, HI and W. A Tukey test was used to perform multiple comparisons in all models.

#### Behavioural parameters

All behavioural data (frequency and duration) were analysed by mixed linear model using PROC mixed procedure with random factors utilised to account for multiple records per animal. Firstly, to identify variation in behavioural parameters over the 8 hour journey duration, models were developed using the hour (from 1^st^ to 8^th^) as a fixed factor, with horse and trip (trip 1, trip 2) as random factors. A separate model was developed with each of the behavioural parameters as the outcome. Secondly, to identify behavioural differences during travelling, during the rest stop and when inside the box, models were developed using the situation (travelling, rest-stop, box) as a fixed factor, with horse and trip as random factors. A separate model was developed with each of the behavioural parameters as the outcome. A Tukey test was used as for *post-hoc* testing.

#### Clinical parameters

Clinical parameters evaluated were derived from clinical examination and clinical pathology (haematology, serum biochemistry, arterial blood gas analysis). Based on the results of lung auscultation at unloading, horses were split into two groups: abnormal lung sounds (AB) and normal (N). The effect of transportation, group (AB or N) or their interaction on these parameters was analysed by mixed linear model using PROC mixed procedure with time (preloading, unloading, AJ), group (AB or N) and their interaction as fixed factors. In the model, horse and trip were included as random factors, to account for multiple observations. A separate model was developed with each clinical parameter as the outcome and *post-hoc* testing was performed by Tukey test.

#### Respiratory parameters

The observations made during endoscopy and TW scores were analysed by ordinal logistic regression analysis with the time, group (AB or N) and their interaction as fixed factors. The horse and trip were used as random factors. The null hypothesis was that the distribution of the scores was the same.

TW data (differential cells count and concentration of bacteria CFU/ml) were analysed by mixed linear model using time, group (AB or N) and their interaction as fixed factors. Horse and trip were used as random factors. A separate model was developed with each of the TW parameters as the outcome. Tukey test was used as a *post-hoc* test. The effect of time on the bacteria DNA concentration was determined by Kruskal-Wallis test.

Statistical analysis of 16s rRNA sequencing data was undertaken using *phyloseq* [43] and *edgeR* [44] packages for R, available through Bioconductor [45]. Taxonomic assignments and associated sample data were imported into R to create a *phyloseq* object. Bacterial diversity changes were evaluated by comparison of the Shannon diversity index, calculated after exclusion of OTUs for which the variance across all samples was very low, at different times (preloading, unloading, 24 hours AJ, 5 days AJ) and between groups (AB and N) by Welch two sample t-test.

Identified bacteria were classified in 5 groups: the family *Pasteurellaceae*, the family *Streptococcaceae*, the family *Enterobacteriaceae*, anaerobes (based on genera associated with equine respiratory disease: *Fusobacterium* spp, *Clostridium* spp, *Peptostreptococcus* spp, *Bacteroides* spp, *Porphorymonas* spp, *Prevotella* spp)[46], and saprophytes. Bacteria which could not be classified into one of these 5 groups were placed in a group designated ‘others’ and pooled within the saprophyte grouping. These data were expressed as a percentage of total amplicons and analysed by mixed linear model using time (preloading, unloading, 24 hours AJ and 5 days AJ), group (AB, N) and their interaction as fixed factors. Horse and trip were included as random factors. A separate model was developed for each bacterial grouping and Tukey was used as a *post-hoc* test. Results are presented as least square mean ± SE.

#### Associations between behavioural, clinical, haematological, and respiratory parameters

Associations between behavioural, clinical, haematological respiratory parameters were explored using Spearman correlation. Associations with a significant spearman Spearman correlation (P<0.05) were further investigated using linear regression analysis. Respiratory parameters (endoscopic scores and TW bacterial concentration, CFUmL) were expressed as the difference between endoscopic scores and TW bacterial concentration (log CFU/mL).

## Results

### Clinical parameters

All horses were normal on clinical evaluation before travelling. Clinical examination findings at unloading are provided in S4 Table. One horse (H10) sustained a hindlimb laceration during transportation which required treatment with antibiotics and anti-inflammatory medication. As this treatment potentially influenced outcome variables in this study, data from this horse were excluded from statistical analysis. Transportation had a significant effect on HR, RR, RT and BW (S5 Table). Ten of twelve horses had decreased borborygmi in one or more quadrants on abdominal auscultation immediately after unloading. Coarse airway sounds were audible during thoracic auscultation for six horses at this time, and three of these horses coughed during the examination. Coughing and coarse airway sounds persisted in these horses until 5 days AJ, however no horse developed pyrexia at any time during the study. Horses with abnormal findings on thoracic auscultation were differentiated as Group AB, with remaining (normal) horses forming Group N. There was no difference between groups for any clinical parameter, and the group*time interaction was also not significant.

### Environmental parameters and air quality inside the truck and the boxes

Environmental temperature ranged from 10°C to 27°C in the boxes and from 10°C to 20°C in the truck during transport, with the highest values recorded during the afternoon. Humidity ranged from 21% to 82% inside the boxes and from 28% to 85% during transport, and was higher in the morning than the afternoon. The heat index ranged from 10°C to 25°C in the boxes and from 10°C to 20°C during transport, increasing progressively during the day. The velocity of air movement ranged from 0 to 5.1 km/h in the moving truck, whilst no air movement was recorded during the rest stop. No air movement was registered inside the box. Mean values for temperature, humidity and heat index varied significantly between boxes and trips (all P<0.001, data not shown). Gas analysis demonstrated oxygen concentration of 20.9 vol% in both boxes and throughout both trips, and NH_3_, H_2_S, CO, CH_4_ gases were not detected.

Bacterial concentrations in air samples from the truck were 3.1 log_10_CFU/m^3^ at preloading and 3.2 log_10_CFU/m^3^ at unloading in trip 1, and 6.2 log_10_CFU/m^3^ at preloading and 4.0 log_10_CFU/m^3^at unloading in trip 2. The bacterial load inside the stable was 5.9 log_10_CFU/m^3^ on the day before travel and 6.1 log_10_CFU/m^3^ after the journey.

### Behavioural parameters

One horse (H3) showed a locomotory stereotypy (head tossing) after the second hour of travel and H2, which was on the right side of H3, started showing the same stereotypy in the last two hours of the trip. During the 7^th^ hour of the journey, H11 showed many aggressive behaviours toward H10, which started kicking in response and was subsequently injured.

#### Effect of the situation (travelling, rest-stop, stable box)

Balance related behaviours, particularly leaning on partitions or loss of balance, were exhibited most often during the journey, compared to in the truck at rest (P<0.001) or in the stable (P = 0.004, Table 2). Backward and forward movements were observed more frequently whilst horses were inside the box than during the journey or rest stop. Lateral movements were more frequent during the journey, occurring with equal frequency in the truck at rest as in the stable. Stress related behaviours including explorative behaviour, sniffing, licking, chewing and putting their nose outside their bay, were observed more when the horses were travelling than at rest (P = 0.044) or in their boxes (P<0.001), whereas pawing and yawning were observed most frequently when the vehicle was stationary. During the rest stop, the horses demonstrated more stressed-related behaviours (P = 0.011), but moved less (P = 0.016) in comparison with being into their stable boxes. Whilst travelling, head down duration was on average approximatively two and four times shorter compared with the rest stop and the stable, respectively (Table 2).

**Table 2:** Effect of the situation (journey, rest stop, stable box) on horse behaviour.

Behavioural Parameters	Journey	Rest stop	Stable box	P
Evasive behaviour/Pulling back	1.3±0.8	0.5±0.9	0.4±0.9	0.163
Explorative behaviour/Sniffing	25.4±10.1^A^	12.9±10.9^Ba^	0.9±10.9^Bb^	< .0001
Licking/chewing	18.1±5.6^A^	12.8±7.0	4.1±7.0^B^	0.0118
Licking the truck/wall of the box	3.1±0.9^a^	3.8±1.5^a^	0.1±1.4^b^	0.0493
Nose outside	15.1±4.8^A^	6.6±6.7	0.0±6.7^B^	0.0097
Pawing	2.1±0.9^a^	6.5±2.0^b^	0.1±2.0^a^	0.0481
Touching rubber tie cord	24.1±3.1	18.1±4.9	na	0.1814
Turning the head	6.5±1.7	2.3±2.8	3.8±2.8	0.1658
**Total stress-behaviours**	**95.6±25.5**^**Aa**^	**63.3±25.4**^**b**^	**9.0±25.4**^**Bc**^	**< .0001**
Backward movements	1.3±0.7^Aa^	0.3±0.8^Ab^	3.1±0.8^B^	< .0001
Forward movements	0.9±1.2^A^	0.6±1.9^A^	21.2±1.9^B^	< .0001
Lateral movements	16.8±2.7^A^	8.1±3.6^B^	8.2±3.7^B^	0.0003
Leaning on stall rails	29.8±3.4^A^	4.2±5.0^B^	na	< .0001
Loss of balance/dashing on the partitions	5.1±1.5^A^	0.2±1.8^B^	na	< .0001
**Total balance related behaviour**	**53.9±4.4**^**A**^	**13.3±6.8**^**Ba**^	**32.8±6.8**^**Bb**^	**< .0001**
Head tossing/shaking	52.4±39.1	30.3±44.7	0.1±44.7	0.089
Biting neighbours	2.4±1.02	0.3±1.8	0.0±1.77	0.167
Interaction with neighbours	34.4±4.6^A^	20.7±6.1^B^	3.4±6.1^C^	< .0001
Looking outside	35.9±4.0^A^	19.8±5.3^B^	11.8±5.3^B^	< .0001
Shaking head	4.9±1.8	3.3±2.6	1.5±2.6	0.2748
Yawning	0.6±0.3^A^	4.0±0.7^B^	0.1±0.7^A^	< .0001
Lowering the head	37.6±3.4^Aa^	31.8±5.4^b^	16.4±5.4^B^	0.0001
**Total movements**	**316.3±58.1**^**A**^	**185.3±62.5**^**B**^	**73.3±62.5**^**C**^	**< .0001**
**Total head down duration**	**273.9±63.5**^**A**^	**644.9±80.9**^**B**^	**1204.7±80.9**^**C**^	**< .0001**

Effect of the situation (journey, rest stop, stable box) on frequency of the measured behavioural events (n/25 min) and total head down duration (s/25 min). Data are expressed as the least square mean and standard error (SE), with P value determined by linear mixed model and Tukey post-hoc testing. Means with different superscript differ significantly (A, B, C P<0.001; a,b P<0.05)

#### Effect of journey duration

The frequency of stress related behaviours was greatest in the first hour of travel and tended to decrease with journey duration (Table 3). Leaning on stall rails was the balance related behaviour most frequently exhibited during transport. Total balance related behaviours decreased until the 5th hour of the trip before increasing to peak during the final hour of travel (Table 3). The frequency with which horses lowered their heads increased during the journey, as did the amount of time spent with in a head down position (Table 3).

**Table 3:** Effect of the journey hour (from the first to the eighth) on horse behaviour.

**Behavioural Parameters**	**1**^**st**^ **h**	**2**^**nd**^ **h**	**3**^**rd**^ **h**	**4**^**th**^ **h**	**5**^**th**^ **h**	**6**^**th**^ **h**	**7**^**th**^ **h**	**8**^**th**^ **h**	**SE**	**P**
Evasive behaviour/Pulling back	2.7	2.2	1.0	1.2	0.3	1.4	0.9	0.6	1.0	0.054
Explorative behaviour/Sniffing	34.9	27.4	25.5	22.9	26.7	17.6	25.6	21.9	12.4	0.228
Licking/chewing	42.9^A^	22.6^Ba^	17.5^B^	12.3 ^B^	15.5 ^B^	10.1 ^Bb^	12.3 ^Bb^	10.7 ^Bb^	7.4	< .0001
Licking the truck/wall	7.0	3.5	2.4	2.8	2.7	2.4	2.3	1.2	1.4	0.067
Nose outside	41.8^A^	24.9^Ba^	10.2^BCb^	15.5^BC^	9.9^BCb^	9.4^BCb^	6.2^C^	2.7^C^	6.7	< .0001
Pawing	5.5	1.9	1.3	3.4	0.8	1.2	1.8	1.1	1.4	0.284
Touching rubber tie cord	35.5^Aa^	30.0^ab^	28.3	21.2^bc^	21.2^bc^	20.8^bc^	18.7^B^	16.9^Bc^	5.2	0.032
Turning the head	12.8	8.4	8.4	5.4	5.0	1.9	4.5	5.9	3.1	0.089
**Total stress-behaviours**	**221.8**^**A**^	**155.7**^**Ba**^	**129.1**^**B**^	**123.3**^**B**^	**117.2**^**B**^	**98.7**^**B**^	**110.0**^**B**^	**94.4**^**Bb**^	**26.8**	**< .0001**
Backward movements	1.6	1.8	1.2	1.6	1.4	1.1	0.9	0.7	0.8	0.773
Forward movements	1.8	1.03	0.5	0.9	0.8	0.7	0.4	0.6	0.6	0.113
Lateral movements	16.1	18.4	14.0	15.3	16.3	15.9	16.5	22.3	3.9	0.343
Leaning on stall rails	34.0^ac^	23.4 ^ab^	26.3 ^ab^	31.5	22.7^b^	30.0 ^ab^	30.1	40.1^c^	5.5	0.023
Loss of balance/ dashing on the partitions	8.8^Aa^	5.6^b^	2.3^BC^	3.6^BC^	4.2 ^BC^	4.7 ^BC^	5.5^b^	6.5^AC^	2.1	0.004
**Total balance related behaviour**	**62.2**^**ac**^	**49.9**^**A**^	**44.1**^**Ab**^	**52.7**^**ab**^	**45.2**^**Ab**^	**52.2**^**ab**^	**53.3**^**ab**^	**70.8**^**Bc**^	**6.8**	**0.006**
Head tossing/shaking	13.6	43.4	46.0	69.9	53.6	77.9	71.7	42.8	49.1	0.428
Biting neighbours	2.6	3.2	1.4	1.3	2.4	0.9	6.1	2.0	1.9	0.451
Interaction with neighbours	43.7	35.9	32.3	31.1	35.4	35.4	32.4	28.9	6.7	0.361
Look outside	38.8	35.0	34.7	38.6	35.1	34.1	38.0	33.4	5.9	0.933
Shaking head	7.0	6.4	3.5	6.4	3.1	3.4	5.7	3.5	2.9	0.779
Yawning	1.3	0.7	1.0	0.8	0.1	0.2	1.0	0.1	0.6	0.672
Lowering the head	12.1^A^	39.7^B^	43.1^Ba^	44.6^Ba^	31.4^Bb^	38.4^B^	42.3 ^Ba^	49.3 ^Ba^	5.0	< .0001
**Total movement**	**362.3**	**333.1**	**298.6**	**328.2**	**286.6**	**305.1**	**320.7**	**289.9**	**71.2**	**0.216**
**Total head down duration**	**55.1**^**A**^	**245.4**^**B**^	**318.9**^**BC**^	**268.3**^**BCa**^	**249.7** ^**B**^	**363.9**^**BC**^	**265.9**^**Ba**^	**429.5**^**Cb**^	**81.6**	**< .0001**

Effect of the journey hour (from the first to the eighth) on frequency of the measured behavioural events (n/25 min) and total head down duration (s/25 min). Data are expressed as the least square mean and standard error (SE), with P value determined by linear mixed model and Tukey post-hoc testing. Means with different superscript differ significantly (A, B, C: P<0.001; a, b: P<0.05)

### Haematology, blood biochemistry and arterial blood gases

The effect of transport on haematological and blood biochemistry parameters are shown in S6 Table. Transient increases were observed in neutrophil counts, total protein, albumin, globulins, glucose and lactate at unloading, but these changes did not differentiate horses with abnormal findings on pulmonary auscultation at unloading (Group AB) from remaining horses. Fibrinogen concentrations were mildly increased at 5 days AJ, but serum amyloid A did not increase. Cortisol changed significantly with transportation (P<0.0001), being significantly increased on arrival and transiently decreased 24h AJ. Creatinine kinase was increased for at least 48 following transportation, with peak values observed at 12h AJ. Potassium, ionised calcium and bicarbonate were all transiently decreased at unloading. There were no effects on arterial blood gas partial pressures or pH (data not shown).

### Respiratory parameters

#### Endoscopic findings

**Fig 1:**
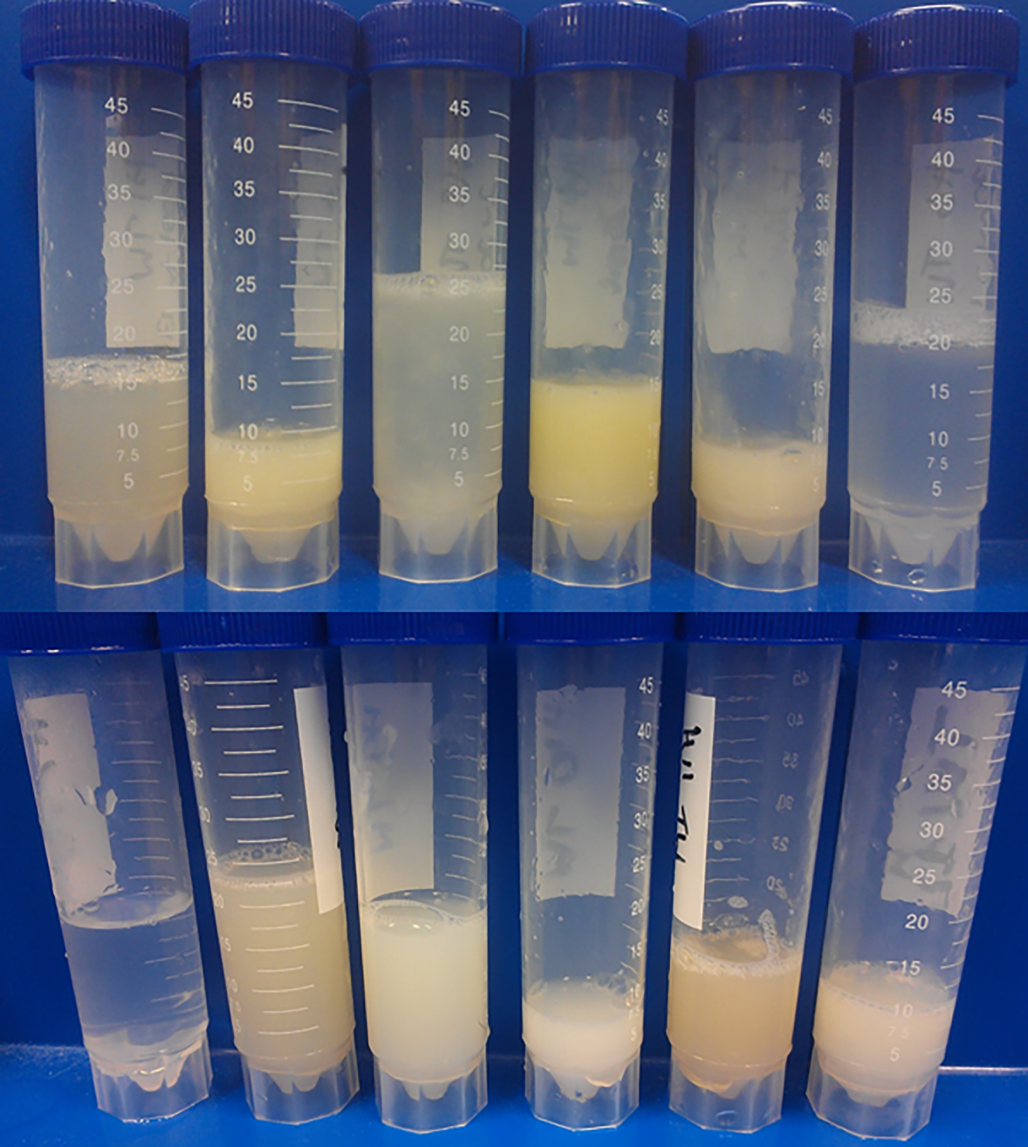
Gross characteristics of endoscopic tracheal wash samples from horses following trip 1 (top) and trip 2 (bottom).

A highly significant group*time interaction was observed for tracheal inflammation as Group AB horses had significantly higher scores than Group N at preloading (P = 0.017), unloading (P = 0.019) and 5 days AJ (P = 0.003). The effect of time of collection and group (AB or N) was significant on tracheal mucus which was lower at preloading in comparison with all the other times (S1 Fig) and higher in group AB in comparison with group B (S1 Fig). TW samples prior to transportation were clear and transparent, whereas those obtained following transportation were white, cream or yellow in colour (Fig 1). The effect of time of collection was indeed significant on both TW colour (S2 Fig) and TW turbidity (S3 Fig) which were increased at unloading compared with preloading and did not restore after 5 days.

#### TW cytology

Both time (P<0.001) and group (AB or N, P = 0.030) had a significant effect on the percentage of neutrophils in TW samples, and the time*group interaction was also significant (P = 0.012). The percentage of neutrophils was highest in TW obtained 24 hours after travel (preloading: 29.68 ± 6.27; unloading: 19.82 ± 6.27; one day AJ: 72.99 ± 6.27; 5 days AJ: 57.65 ± 6.27%). Results from Group N had returned to pre-transportation values by Day 5 AJ, whereas results from Group AB showed a persistent airway neutrophilia (AB: 83.4 ± 8.4% vs N: 31.9 ± 9.3%; P = 0.001).

#### Phenotypic and quantitative bacteriology of TW samples

**Fig 2:**
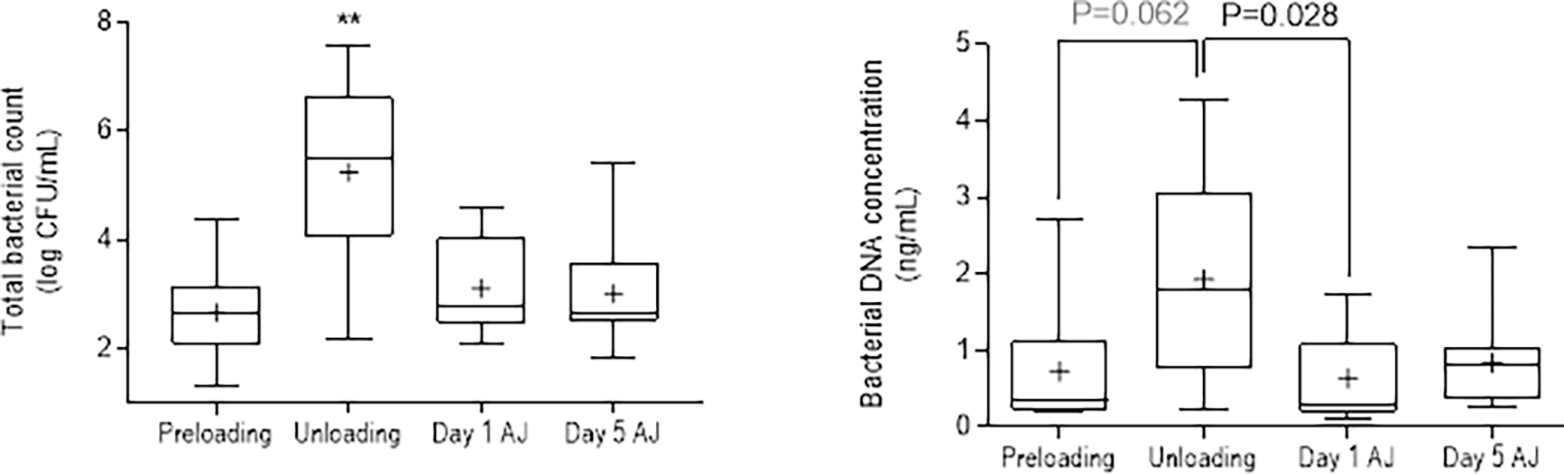
Bacterial load determined by conventional quantitative bacteriology (left) and genomic analysis (right). Results for conventional bacteriology results were compared by analysis of variance after log transformation of results, with significantly increased numbers of bacteria at Unloading relative to all other times (**, P<0.001). Results from genomic analysis were compared by Kruskal-Wallis test, with results where P<0.1 shown. CFU = colony forming units; AJ = after journey.

The time had a significant effect on the concentration of bacteria recovered from TW (P<0.001, Fig 2). At unloading there was an increase of approximately 10^3^ colony forming units (CFU) / mL in both groups, which was significant when compared with all other sample times (P<0.001). Results from Group AB (abnormal pulmonary auscultation) had a higher overall concentration of bacteria across all sampling times than was observed for group B (P = 0.013). The effect of the time*group interaction was not significant (P = 0.701). At unloading the predominant bacterial isolates were identified as *Pasteurellaceae*-like (i.e. Gram negative, oxidase positive rods) for the majority of horses (n = 8).

#### Bacteriology microbiome

**Fig 3:**
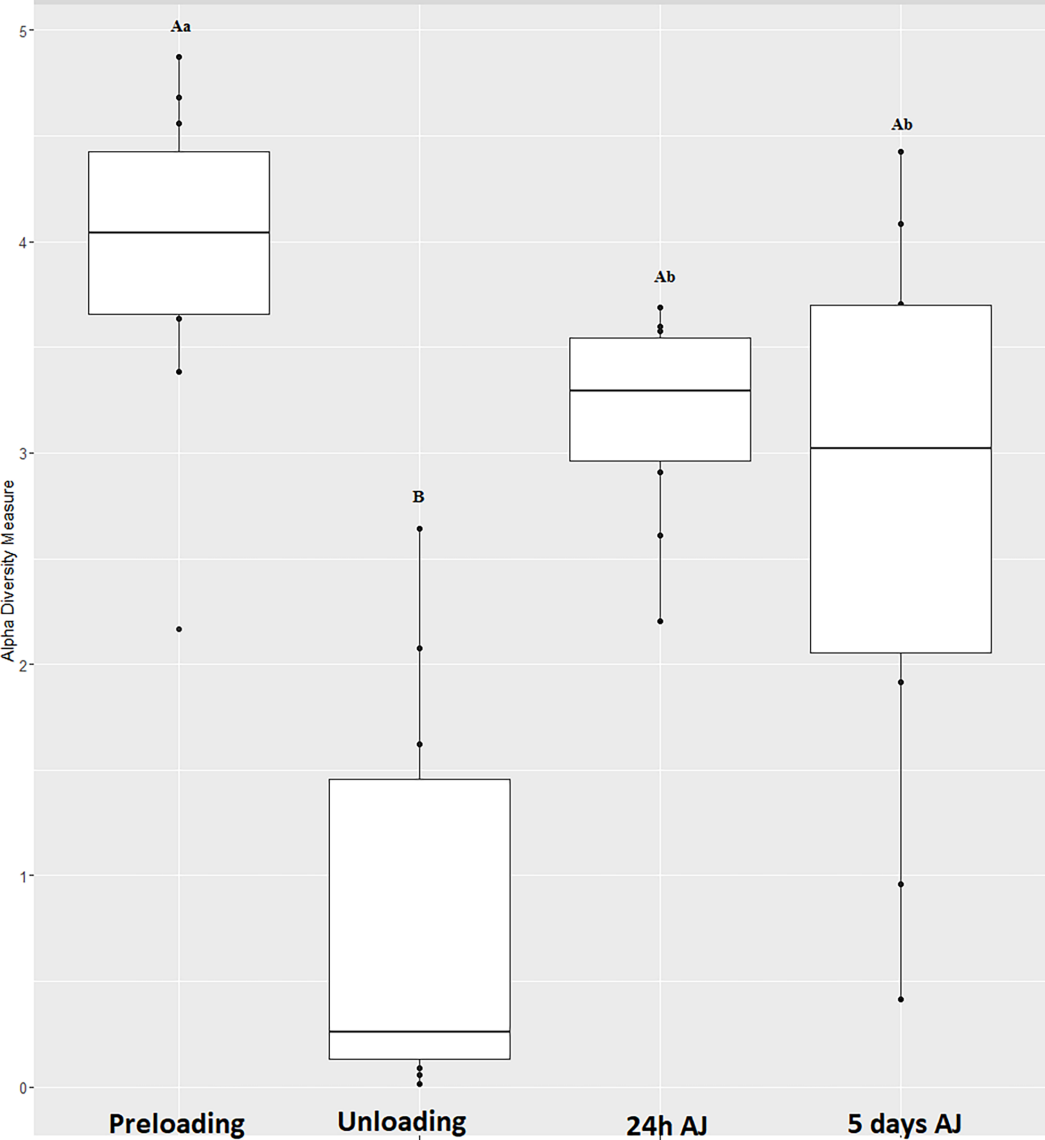
Effect of time on Shannon diversity index.

The quantity of DNA extracted for analysis from TW increased at unloading, as was observed for conventional quantitative bacterial counts (Fig 2). There was a significant decrease in the Shannon diversity index (Fig 3) between preloading and unloading, reflecting a significant reduction in diversity of respiratory flora induced by the act of transportation. The microbiome diversity did not restore with a significance difference seen in microbiome diversity when comparing preload and 24h and 5 days AJ.

**Fig 4:**
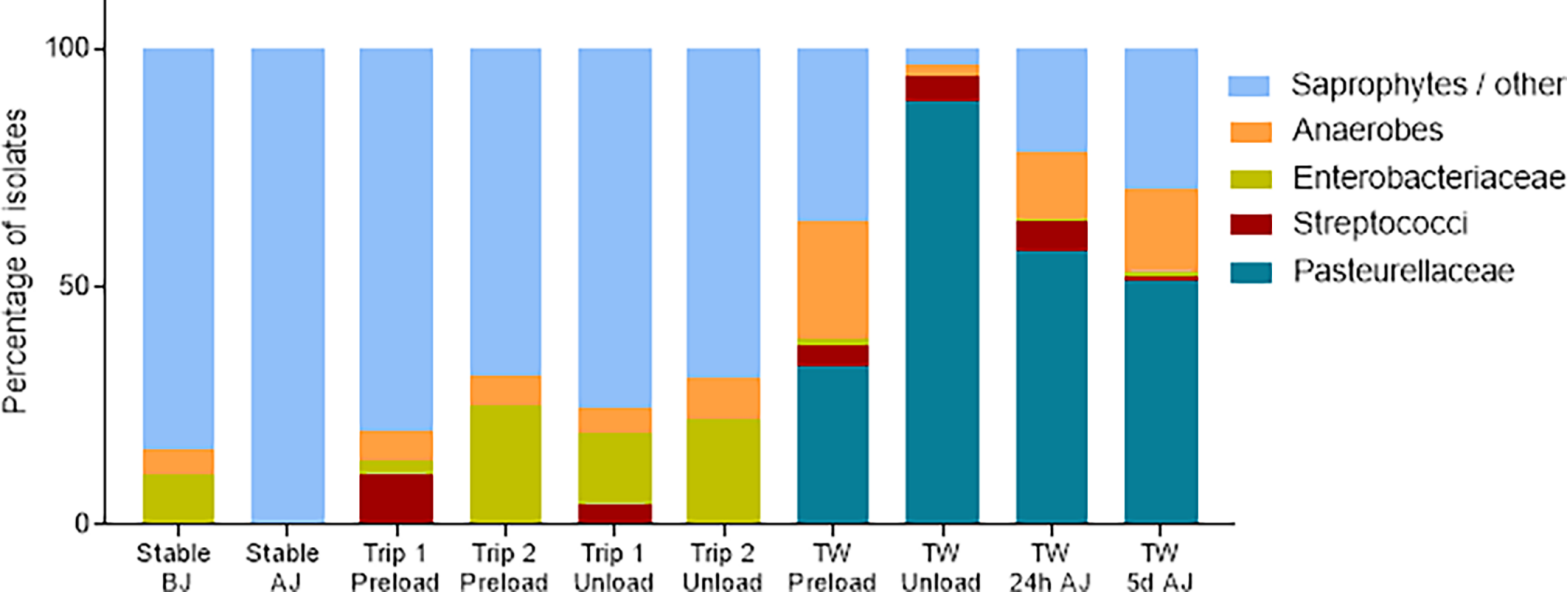
Effect of transportation on tracheal wash microbiome in comparison with air sample microbiome. Results are presented as the relative proportion of organisms present in air samples collected in stable and truck before and after journey (BJ; AJ) and in TW samples collected at preloading, at unloading, and 1 and 5 days after travel. Note that the absolute TW bacterial load increased immediately following transportation.

Time of sampling had a significant effect bacterial species identified in TW samples, and species identified in TW samples were different to those present in air samples (Fig 4). Bacterial isolates in environmental air samples were predominantly from the families *Corynebacteriaceae*, *Yaniellaceae* and *Staphylococcaceae*, whereas TW samples included *Pasteurellaceae*, with this taxa dominating the respiratory flora of many horses at unloading. As was observed with phenotypic methods, *Pasteurellaceae*-like bacteria were identified with increased frequency in samples collected at unloading, whereas the relative abundance of strict anaerobes and saprophytes decreased in the total TW microbiome at this time (P<0.001)(Fig 4). Genera identified within the *Pasteurellaceae* included *Aggregatibacter* and *Actinobacillus* spp. The relative abundance of *Streptococcaceae* and *Enterobacteriaceae* did not change with time, and there was no difference in the relative abundance of different organisms between AB and N horses.

### Spearman correlation and linear regression analysis: associations between behavioural and clinical parameters

The amount of time that horses spent with their head down was negatively associated with the tracheal mucus and inflammation scores and the TW bacterial concentration (log_10_ CFU/ml), but also with stress-related behaviours such as licking/chewing and pawing. Heavier animals showed more licking/chewing behaviours and spent less time with their heads down. On review of video footage it was apparent that that some larger framed horses were physically unable to lower their heads in the available space. The animals that moved more *en route* showed higher plasma concentrations of total protein and CK on unloading. There was an association between objective indicators of stress at unloading (plasma cortisol concentration and HR) with behaviours interpreted as indicative of stress such as touching the rubber tie cord, evasive behaviours. HR at unloading was also associated with balance related behaviours such as forward and backward movements. The animals which showed more stress related behaviours *en route* also had higher serum fibrinogen concentrations at unloading. The total white cell count was positively correlated with fibrinogen and globulins (Table 4). No other significant association/correlations were observed.

**Table 4:** Associations between behavioural, clinical, haematological and respiratory parameters.

X	Y	r	P^a^	R^2^	P^b^
Total head down duration	Tracheal mucus score	-0.717	0.012	46.1	0.013
	TW Bacteria log10CFU/ml	-0.686	0.019	41.3	0.020
	Tracheal inflammation score	-0.613	0.044	30.7	0.045
	Licking/Chewing	-0.718	0.012	46.2	0.013
	Pawing	-0.637	0.035	34.0	0.035
Total Movements	CK	0.658	0.027	36.9	0.028
	TP	0.619	0.042	31.5	0.042
Touching rubber tie cord	Cortisol	0.724	0.0117	61.5	0.012
	Tracheal inflammation	0.679	0.021	40.2	0.021
	Lowering the head frequency	-0.725	0.011	47.4	0.011
Total stress related behaviours	Fibrinogen	0.648	0.031	35.5	0.031
HR	Backward movement	0.914	<0.001	81.8	<0.001
	Forward movements	0.729	0.010	48.0	0.011
	Evasive behaviours	0.808	0.002	61.5	0.003
White blood cell	Neutrophils	0.927	<0.001	84.5	<0.001
	Fibrinogen	0.773	0.005	55.4	0.005
	Globulin	0.684	0.020	40.9	0.020
	Albumin	-0.757	0.007	52.7	0.007

Pearson correlations and linear regression analysis results showing associations between explanatory (X) and outcome (Y) factors. Data expressed as r and P^a^ for the correlation, and as R^2^ and P^b^ for the linear regression.

## Discussion

This multidisciplinary study replicated previously reported haematological, serum biochemistry and respiratory effects of transportation and, for the first time, related these changes to observed behavioural responses to transportation, supporting our hypothesis. Heart rate and serum cortisol concentrations were higher at unloading (evening) than at preloading, suggesting that transportation is a mental and physical stressor for horses, particularly as circadian secretion of cortisol in horses typically results in higher concentrations in the morning than evening [47]. As both stress-indicators were restored to normal values within 12 hours after the journey, the transport event in the current study should be considered an acute stressor with relatively rapid recovery.

The frequency of stress-associated behaviours was correlated to serum cortisol concentrations at unloading and horses that exhibited stress-associated behaviours more frequently also spent more time with their heads in an elevated position *en route* and during the rest stop, accumulating more mucus and bacteria in their lower respiratory tract. As such, recognition of these behaviours might allow pre-emptive identification of individual animals at increased risk for respiratory disease. The highest frequency of stress related behaviours was registered during the first hour of transport, suggesting this to be the most stressful part of a journey. Similar findings have been made in other studies using either cortisol or other stress indicators, such as heart rate variability, thyroid and adrenocortical hormones [27, 48–50]. Based on our study and the findings of others, it seems that horses adapt to the journey and to the vehicle after approximately 5 hours. However, our behavioural responses might also indicate fatigue, evidenced by increased movements relating to balance after the fifth hour of transport, the observed peak in balance related behaviour at the 8^th^ hour, increased lactate and CK at unloading. Individual differences were noticed in behavioural responses, with the more nervous and agitated horses affecting their neighbours. As social animals, anxiety and arousal can be socially transmitted to herd mates [29, 51], a factor that should be taken into account when mixing horses with different temperament and travel experiences.

The transport related changes in respiratory microbiota at unloading are in agreement with the literature [1, 52] but, for the first time, results of conventional quantitative bacteriology are juxtaposed with characterisation of the TW microbiome. Conventional and molecular methods demonstrated an increased bacterial load in the majority of horses immediately after transportation, associated with a preferential multiplication of *Pasteurellaceae* bacteria, as previously reported [1, 52]. Comparison of equine respiratory and equine environmental microbiomes demonstrated that the lower respiratory tract is invaded mainly by oropharyngeal commensal organisms and not by environmental bacteria. These findings support the role of *Pasteurellaceae* as an early, opportunistic invaders when pulmonary clearance mechanisms are compromised [12]. There was no evidence of concurrent increases in other bacteria previously associated with pleuropneumonia in horses in this study, such as *Enterobacteriaceae*, *Streptococcus* spp or strict anaerobes, suggesting that these organisms may become part of the disease process at a later stage or under different circumstances.

Examination of TW samples showed a return to near normal (preload) concentrations of respiratory bacteria 24 hours AJ along with a return to a more diverse microflora community. However tracheal inflammation and mucus, TW colour and turbidity scores in our horses did not recover in the day after the journey, as had been demonstrated in previous studies [1, 52], a discrepancy that might be related to differences in management post journey. In the above mentioned studies horses were kept on pasture while our horses were stabled. Despite being open on all four sides, the air quality in these stables was relatively poor (> 3 log_10_ CFU/m^3^) and stabled horses have been reported to spend less time with their heads down [53]. Further studies are needed to determine whether keeping horses on pasture for 24 hours after a journey promotes clearance of inflammatory secretions and bacteria following transportation.

Pulmonary auscultation of horses at unloading identified six horses (Group AB) with increased airway sounds, three of which were also observed coughing. This group subsequently proved to have increased airway inflammation and secretions, increased airway neutrophilia and concentration of bacteria recovered from TW samples compared to the remaining horses. As these animals’ tracheal inflammation scores prior to transportation were also higher than Group N horses, these horses may have been at increased risk for airway bacterial proliferation and contamination due to sub-clinical airway inflammation. These observations emphasise the importance of thorough veterinary examination before and after transportation.

Transportation was associated with an acute phase inflammatory response characterised by transient neutrophilia, increased fibrinogen and increased globulins, as has been previously observed [19]. Mild, but significative, increases were observed in albumin, total protein, capillary refill time and body weight decreased, all consistent with decreased total body water [54]. Horses in the current study travelled without food or water for 8 hours but within the thermo-neutral zone, which is between 5°C and 25°C [55] and in absence of noxious gases [56, 57], which might account for the physiologically minor changes in fluid balance observed. Despite their modest magnitude, the observed changes in albumin, protein and body weight were not restored within 24 hours, suggesting that recovery from transportation might require a number of days. More severe changes might be expected with journeys of longer duration or under more extreme environmental conditions.

Systemic electrolyte changes, specifically reduced plasma K^+^ and Ca^++^, were observed transiently following transportation. Decreased plasma K^+^ has been previously reported [19] and might be due to fasting [58] or transcellular movement of K^+^. Transportation has been suggested as a predisposing factor for the development of hypocalcaemic tetany [59], however, to the authors’ knowledge, to date there has been limited evidence of decreased ionised calcium in horses subsequent to transportation [60]. Blood lactate and CK were mildly elevated at unloading, and plasma CK concentrations were related to the frequency of movement and balance behaviours, confirming that the continuous movements *en route* affected the musculoskeletal system. Our horses were rested in their home stable following their journey, but lactate and CK remained elevated for a time AJ, suggesting a period of 24 hours rest after journeys of similar or greater duration should be recommended.

Limitations of the current study include the relatively small number of research horses, which may not have been as well conditioned to transportation as performance horses. However, the study was adequately powered for key outcome variables, and previous studies have used the same or smaller sample size. As even experienced horses still show a pronounced stress response to long distance transportation [61], these results are likely applicable to horses transported in other settings. Transportation occurred on two different days, which were not identical for environmental parameters. However, ‘trip’ was included as an explanatory variable in data analysis to account for these differences. Physiological parameters, heart rate, respiratory rate and temperature, could not be recorded *en route*, and the monitoring system used did not record sounds, so we could not measure noise inside the truck nor assess vocalizations, which is recognised as a stress indicator in horses [62]. Notwithstanding these limitations, this is the first study to relate behavioural changes during transportation to subsequent lower airway bacterial proliferation, contamination and inflammation.

## Conclusions

Overall, this multidisciplinary study showed that the level of stress experienced by the animal *en route* is manifested with increased frequency of stress-related behaviours and reduced duration with their head in the downward position. The elevated head position correlated with the accumulation of tracheal mucus and increased numbers of respiratory bacteria, with prominence of commensals within the *Pasteurellaceae* family. From our findings, accurate clinical examination including auscultation prior to and after journey, monitoring of horse behaviours *en route* and 24 hour rest AJ are recommended to reduce the incidence of transport-related respiratory disease and to identify the animals at risk of these disease.

## Supporting information

S1 Table Experimental protocol. (DOCX)

S2 Table Clinical sheet recording observations during respiratory endoscopy. (DOCX)

S3 Table Bacterial isolates. Working definitions for phenotypic identification of bacterial isolates from tracheal wash samples (from [12]).
(DOCX)

S4 Table Clinical examination results at unloading. (DOCX)

S5 Table Effect of transportation on heart rate (HR), respiratory rate (RR), rectal temperature (RT) and body weight (BW). (DOCX)

S6 Table Effect of transport on haematology, blood biochemistry and arterial blood gases. (DOCX)

S1 Fig Effect of the Time (a) and Group (b) on the distribution of the Tracheal Mucus score (0 = none, 1 = little, 2 = moderate, 3 = marked, 4 = large, 5 = extreme).
(DOCX)

S2 Fig Effect of the time on the distribution of the TW colour score (1 = clear, 2 = white, 3 = yellow, 4 = blood). (DOCX)

S3 Fig Effect of the time on the distribution of the TW turbidity score (0 = transparent, 1 = clouded, 2 = smoked glassed, 3 = opaque). (DOCX)

S1 Appendix Minimal data set. (XLSX)
