# Supplementary material for: Behaviour during transportation predicts stress response and lower airway contamination in horses
Source: PLoS One. 2018 Mar 22;13(3):e0194272. doi: 10.1371/journal.pone.0194272 (PMC5863983; doi:10.1371/journal.pone.0194272)
Supplement: S1 Table — (DOCX) [file pone.0194272.s001.docx]

**S1 Table. Experimental protocol.**

|  | **Day** | 1 | 2-8 | 9-15 | 16 | 17 | 18 | 19 | | | 20 | | 21 | | | 22 | | 23 | 24 | 25 | 26 | 33 | 35 |
| --- | --- | --- | --- | --- | --- | --- | --- | --- | --- | --- | --- | --- | --- | --- | --- | --- | --- | --- | --- | --- | --- | --- | --- |
|  | **Hours** |  |  |  |  |  |  | 6.00 |  | 17.00 | 6.00 | 17.00 | 6.00 | | | 6.00 | | 6.00 | 6.00 |  | | 6.00 |  |
| Group 1 | CEx | ✓ | First habituation week | Second habituation week | ✓ |  |  | ✓ | Travel | ✓ | ✓ | ✓ | ✓ | | | ✓ | | ✓ | ✓ |  | |  |  |
|  | Ven B | ✓ |  |  |  |  |  | ✓ |  | ✓ | ✓ | ✓ |  | | |  | |  | ✓ |  |  | ✓ |  |
|  | Art B |  |  |  |  |  |  | ✓ |  | ✓ |  | ✓ |  | | |  | |  | ✓ |  |  |  |  |
|  | Weight | ✓ |  |  |  |  |  | ✓ |  | ✓ |  | ✓ |  | | |  | |  | ✓ |  |  |  |  |
|  | Scop+TW |  |  |  | ✓ |  |  |  |  | ✓ |  | ✓ |  | | |  | |  | ✓ |  |  |  |  |
|  | **Hours** |  |  |  |  |  |  |  | | |  | | 6.00 |  | 17.00 | 6.00 | 17.00 | 6.00 | 6.00 | 6.00 | 6.00 |  | 6.00 |
| Group 2 | CEx | ✓ | First habituation week | Second habituation week |  |  |  |  | | |  | | ✓ | Travel | ✓ | ✓ | ✓ | ✓ | ✓ | ✓ | ✓ |  |  |
|  | Ven B | ✓ |  |  |  |  |  |  | | |  | | ✓ |  | ✓ | ✓ | ✓ |  |  |  | ✓ |  | ✓ |
|  | Art B |  |  |  |  |  |  |  | | |  | | ✓ |  | ✓ | ✓ | ✓ |  |  |  | ✓ |  |  |
|  | Weight | ✓ |  |  |  |  |  |  | | |  | | ✓ |  | ✓ |  | ✓ |  |  |  | ✓ |  |  |
|  | Scop+TW |  |  |  |  |  | ✓ |  | | |  | |  |  | ✓ |  | ✓ |  |  |  | ✓ |  |  |

Detailed experimental protocol; same procedures were undertaken on each group (n=6) 48 hours apart.

CEx: Clinical examination; Ven B: Venous blood; Art B: Arterial blood; Scop: Scoping; TW: Tracheal wash.
