# Supplementary material for: Behaviour during transportation predicts stress response and lower airway contamination in horses
Source: PLoS One. 2018 Mar 22;13(3):e0194272. doi: 10.1371/journal.pone.0194272 (PMC5863983; doi:10.1371/journal.pone.0194272)
Supplement: S2 Table — (DOCX) [file pone.0194272.s002.docx]

**S2 Table.** **Clinical sheet recording observations during respiratory endoscopy.**

| \| **Tracheal Inflammation: 0** No **1** Mild **2** Moderate **3** Extreme  **Tracheal Mucus:**  **Quantity**   \| **0** \| None present – clean \| \| --- \| --- \| \| **1** \| Little – multiple small blobs \| \| **2** \| Moderate – larger blobs \| \| **3** \| Marked – Confluent stream-forming \| \| **4** \| Large – pool forming \| \| **5** \| Extreme – profuse amounts \|  \| **TW Colour:** \| \| \| --- \| --- \| \| **1** \| Clear \| \| **2** \| White \| \| **3** \| Yellow \| \| **4** \| Blood \|  \| **TW Turbidity:** \| \| \| --- \| --- \| \| **0** \| Transparent \| \| **1** \| Clouded \| \| **2** \| Smoked Glass \| \| **3** \| Opaque \| \| \| \| --- \| --- \| --- \| --- \| --- \| --- \| --- \| --- \| --- \| --- \| --- \| --- \| --- \| --- \| --- \| --- \| --- \| --- \| --- \| --- \| --- \| --- \| --- \| --- \| --- \| --- \| --- \| --- \| --- \| --- \| --- \| --- \| --- \| --- \| \|  \| \| \|  \| \| |
| --- | --- | --- | --- | --- | --- | --- | --- | --- | --- | --- | --- | --- | --- | --- | --- | --- | --- | --- | --- | --- | --- | --- | --- | --- | --- | --- | --- | --- | --- | --- | --- | --- | --- | --- | --- | --- | --- | --- |
