# Supplementary material for: Behaviour during transportation predicts stress response and lower airway contamination in horses
Source: PLoS One. 2018 Mar 22;13(3):e0194272. doi: 10.1371/journal.pone.0194272 (PMC5863983; doi:10.1371/journal.pone.0194272)
Supplement: S3 Table — Working definitions for phenotypic identification of bacterial isolates from tracheal wash samples (from [12]). (DOCX) [file pone.0194272.s003.docx]

**S3 Table**. **Bacterial isolates.** Working definitions for phenotypic identification of bacterial isolates from tracheal wash samples (from [12]).

| Isolate | Definition |
| --- | --- |
| α-haemolyotic *Streptococcus* spp | Gram positive facultative anaerobic cocci in pairs or chains with obvious α-haemolysis on SBA; oxidase negative, catalase negative |
| β-haemolytic *Streptococcus* spp | Gram positive facultative anaerobic cocci in pairs or chains with obvious β-haemolysis on SBA; oxidase negative, catalase negative |
| non-haemolytic *Streptococcus* spp | Gram positive facultative anaerobic cocci in pairs or chains with no haemolysis on SBA; oxidase negative, catalase negative |
| *Staphylococcus* spp | Gram positive facultative anaerobic cocci in pairs, tetrads, or more often grouped in irregular clusters, catalase positive |
| *Pasteurellaceae* spp | Gram negative, facultative anaerobic pleomorphic coccobacilli or bacilli with characteristic colonial morphology (round, greyish or yellowish, slightly raised and nearly 2mm in diameter after 48hr, often sticky in nature), oxidase positive. |
| *Enterobacteriaceae* spp | Gram negative, facultative anaerobic bacilli, with small grey colonies, oxidase negative. |
