# Supplementary material for: Behaviour during transportation predicts stress response and lower airway contamination in horses
Source: PLoS One. 2018 Mar 22;13(3):e0194272. doi: 10.1371/journal.pone.0194272 (PMC5863983; doi:10.1371/journal.pone.0194272)
Supplement: S4 Table — (DOCX) [file pone.0194272.s004.docx]

**S4 Table. Clinical examination results at unloading.**

|  | **H1** | **H2** | **H3** | **H4** | **H5** | **H6** | **H7** | **H8** | **H9** | **H11** | **H12** | **Normal value** |
| --- | --- | --- | --- | --- | --- | --- | --- | --- | --- | --- | --- | --- |
| **RT (°C)** | 38.0 | 38.2 | 38.2 | 38.5 | 38.1 | 38.1 | 37.4 | 37.7 | 37.9 | 37.6 | 38.1 | 37.0-38.5 |
| **HR(bpm)** | 40 | 46 | 42 | 44 | 40 | 36 | 36 | 44 | 40 | 52 | 52 | 30-40 |
| **RR (bpm)** | 12 | 24 | 12 | 16 | 12 | 16 | 16 | 28 | 20 | 40 | 12 | 8-12 |
| **Loss in BW (%/h)** | 0.31 | 0.26 | 0.34 | 0.56 | 0.40 | 0.25 | 0.08 | 0.29 | 0.72 | 0.22 | 0.32 |  |
| **Lung Sound** | Abn | Abn | Abn | Abn | Nor | Nor | Nor | Nor | Nor | Abn | Abn |  |
| **Left dorsal flank** | 1 | 0 | 0 | 0 | 1 | 1 | 1 | 2 | 2 | 0 | 1 | 2 |
| **Left ventral flank** | 2 | 2 | 2 | 2 | 1 | 1 | 2 | 2 | 2 | 1 | 1 | 2 |
| **Right dorsal flank** | 1 | 0 | 1 | 1 | 1 | 1 | 1 | 2 | 2 | 0 | 1 | 2 |
| **Right ventral flank** | 2 | 1 | 2 | 1 | 1 | 1 | 2 | 2 | 2 | 2 | 1 | 2 |
| **GIT total score** | 6 | 3 | 5 | 4 | 4 | 4 | 6 | 8 | 8 | 3 | 4 | 7/8 |
| **Membrane colour** | pink | pink | pink | pink | pink | pink | pink | pink | pink | pink | pink | pink |
| **Membrane status** | dry | dry | dry | dry | dry | dry | dry | dry | dry | dry | dry | wet |
| **CRT (sec)** | 3 | 3 | 3 | 2.5 | 3 | 3 | 3 | 3 | 3 | 3 | 3 | 1-2 |
| **Demeanour** | quiet | quiet | quiet | quiet | quiet | quiet | quiet | quiet | quiet | quiet | quiet | alert |
| **Coughing** | + | + | + | - | - | - | - | - | - | - | - |  |
| **Lymph nodes** | Nor | Nor | Nor | Nor | Nor | Nor | Nor | Nor | Nor | Nor | Nor |  |

Results of the clinical assessment conducted at unloading according to the methodology modified by Padalino et al {Padalino, 2017 #493}.

H: horse; RT: rectal temperature; HR: heart rate; RR: respiratory rate; CRT: capillary refill time; GIT: gastro intestinal tract; Nor: Normal; Abn: Abnormal; +: positive; -: Negative
