# Supplementary material for: Behaviour during transportation predicts stress response and lower airway contamination in horses
Source: PLoS One. 2018 Mar 22;13(3):e0194272. doi: 10.1371/journal.pone.0194272 (PMC5863983; doi:10.1371/journal.pone.0194272)
Supplement: S5 Table — (DOCX) [file pone.0194272.s005.docx]

**S5 Table. Effect of transportation on heart rate (HR), respiratory rate (RR), rectal temperature (RT) and body weight (BW)**

| Parameter | Preloading | Unloading | 12h AJ | 24h AJ | 5d AJ | SE | P value |
| --- | --- | --- | --- | --- | --- | --- | --- |
| HR (beat/min) | 34.7^A^ | 43.3^B^ | 36.4^A^ | 36.4^A^ | 34.7^A^ | 1.1 | <0.001 |
| RR (breath/min) | 13.2^a^ | 18.9^Bb^ | 13.5^a^ | 12.1^A^ | 11.1^A^ | 1.6 | 0.0045 |
| RT (˚C) | 37.5^A^ | 38.0^B^ | 37.5^A^ | 37.5^A^ | 37.3^A^ | 0.1 | <0.001 |
| Body weight (kg) | 444.0^A^ | 431.2^Ba^ | . | 437.4^Bb^ | 442.9^A^ | 15.8 | <0.001 |

Effect of transportation on heart rate (HR), respiratory rate (RR), rectal temperature (RT) and body weight (BW) at preloading, unloading, 12 and 24 hours after journey (AJ), and at 5 days AJ. Data are expressed as the least square mean and standard error (SE), with P value determined by linear mixed model and Tukey post-hoc testing. Means with different superscripts differ significantly (A, B: P<0.001; a, b: P<0.05)
