# Supplementary material for: Behaviour during transportation predicts stress response and lower airway contamination in horses
Source: PLoS One. 2018 Mar 22;13(3):e0194272. doi: 10.1371/journal.pone.0194272 (PMC5863983; doi:10.1371/journal.pone.0194272)
Supplement: S6 Table — (DOCX) [file pone.0194272.s006.docx]

**S6 Table. Effect of transport on haematology, blood biochemistry and arterial blood gases**

| **Parameter** | **Pre-loading** | **Un-loading** | **12h AJ** | **24h**  **AJ** | **5 d AJ** | **SE** | **P value** | **Normal laboratory range** |
| --- | --- | --- | --- | --- | --- | --- | --- | --- |
| RBC  (x10^12^/L) | 7.6^a^ | 7.2^A^ | 8.0 | 8.5^Bb^ | 7.9 | 0.2 | 0.0006 | 6.5-12.5 |
| Haemoglobin  (g/L) | 116.3^A^ | 113.6^A^ | 121.5 | 130.8^B^ | 121.6 | 3.7 | 0.0009 | 110-190 |
| Haematocrit  (%) | 33.4^a^ | 32.2^A^ | 35.1 | 37.4^Bb^ | 35.1 | 1.0 | 0.0010 | 32-52 |
| WBC  (x10^9^/L) | 7.4 | 8.1 | 7.6 | 8.0 | 6.8 | 0.5 | 0.0768 | 5.5-12.5 |
| Neutrophils  (x10^9^/L) | 4.2^A^ | 6.5^B^ | 4.3^A^ | 4.4^A^ | 3.4^A^ | 0.4 | <0.001 | 2.5-8.0 |
| Lymphocytes  (x10^9^/L) | 2.7^A^ | 1.4^B^ | 2.9 ^A^ | 3.2^A^ | 3.2 ^A^ | 0.3 | <0.001 | 1.5-5.5 |
| Monocytes  (x10^9^/L) | 0.22 | 0.07^a^ | 0.24^b^ | 0.24^b^ | 0.15 | 0.04 | 0.0149 | 0.0-0.9 |
| Eosinophils (x10^9^/L) | 0.23^A^ | 0.01^B^ | 0.05^B^ | 0.12 | 0.10 | 0.03 | 0.0003 | 0.0-0.8 |
| Basophils (x10^9^/L) | 0.04 | 0.00 | 0.02 | 0.06 | 0.02 | 0.02 | 0.2310 | 0.0-0.3 |
| Total Protein (g/L) | 65.1 ^A^ | 69.6 ^B^ | 69.4 ^B^ | 68.3 ^B^ | 64.3 ^A^ | 1.2 | <0.001 | 58-76 |
| Albumin  (g/L) | 28.2^A^ | 30.3^B^ | 29.9^B^ | 29.7^B^ | 28.5^A^ | 0.7 | <0.001 | 28-38 |
| Globulin  (g/L) | 36.9^ACa^ | 39.3^ABb^ | 39.5^B^ | 38.5^AB^ | 35.8^C^ | 1.5 | <0.001 | 26-40 |
| Fibrinogen (g/L) | 3.8^a^ | 3.8^a^ | 3.5^a^ | 3.7^a^ | 5.4^b^ | 0.4 | 0.0087 | 2.0-4.0 |
| SAA  (mg/L) | 1.6 | 2.8 | 2.2 | 2.9 | 2.8 | 0.9 | 0.7537 | < 7 |
| Cortisol  (nmol/L) | 121.4^A^ | 182.1^B^ | 140.7^A^ | 74.5^Cc^ | 115.8^Aa^ | 8.3 | <0.001 | n.a. |
| Creatine kinase (U/L) | 248.8^a^ | 291.4^b^ | 294.5^b^ | 292.5^b^ | 268.5^ab^ | 9.9 | 0.0072 | 50-400 |
| AST  (U/L) | 271.0 | 272.5 | 287.8 | 270.2 | 245.2 | 29.8 | 0.8725 | 150-400 |
| Na^+^  (mmol/L) | 133.20 | 134.20 |  | 133.11 |  | 0.65 | 0.211 | 132 - 142 |
| K+  (mmol/L) | 3.94 ^Aa^ | 3.48 ^B^ |  | 4.23 ^Ab^ |  | 0.08 | <0.001 | 2.9 – 4.6 |
| Ca++ (mmol/L) | 1.51 ^Aa^ | 1.42 ^B^ |  | 1.58 ^Ab^ |  | 0.02 | <0.001 | 1.4 – 1.72 |
| Glucose (mmol/L) | 5.24 ^A^ | 6.71 ^B^ |  | 6.10 ^C^ |  | 0.30 | <0.001 | 3.4- 7.4 |
| Lactate (mmol/L) | 0.51 ^Aa^ | 0.77 ^b^ |  | 0.81 ^B^ |  | 0.09 | 0.002 | < 2.0 |
| Bicarbonate  (mmol/L) | 31.68 ^a^ | 29.51 ^b^ |  | 31.25 ^a^ |  | 0.51 | 0.012 | 26 - 32 |

Effect of the time (Preloading, Unloading, 12h AJ, 24h AJ, 5d AJ) on haematological and blood biochemistry parameters. Electrolytes, glucose, lactate and bicarbonate concentrations were determined from arterial samples: remaining analytes were determined from venous blood. Data are expressed as the least square mean and standard error (SE), with P value determined by linear mixed model and Tukey post-hoc testing. Means with different subscript differ significantly (A, B, C P<0.001; a, b P>0.05)

RBC: red blood cells; Hb: haemoglobin; Hct: hematocrit; WBC: white blood cells N: neutrophils; L: lymphocytes; M: monocytes; E: eosinophils; B: basophils; TP: total proteins; Alb: albumin; Glob: globulins; Alb/Glob: albumin globulins ratio; CK: creatine kinase; AST: aspartate aminotransferase; SAA: serum amyloid A.
