# Supplementary material for: Behaviour during transportation predicts stress response and lower airway contamination in horses
Source: PLoS One. 2018 Mar 22;13(3):e0194272. doi: 10.1371/journal.pone.0194272 (PMC5863983; doi:10.1371/journal.pone.0194272)
Supplement: S1 Fig — Effect of the Time (a) and Group (b) on the distribution of the Tracheal Mucus score (0 = none, 1 = little, 2 = moderate, 3 = marked, 4 = large, 5 = extreme). (DOCX) [file pone.0194272.s007.docx]

**S1 Fig.** **Effect of the Time (a) and Group (b) on the distribution of the Tracheal Mucus score (0=none, 1=little, 2=moderate, 3=marked, 4= large, 5=extreme).**

a

b

b

a

Columns with different superscripts are significantly different: A, B P<0.01; a, b P<0.05.
