# Supplementary material for: Behaviour during transportation predicts stress response and lower airway contamination in horses
Source: PLoS One. 2018 Mar 22;13(3):e0194272. doi: 10.1371/journal.pone.0194272 (PMC5863983; doi:10.1371/journal.pone.0194272)
Supplement: S2 Fig — (DOCX) [file pone.0194272.s008.docx]

**S2 Fig.** **Effect of the Time on the distribution of the TW colour score (1=clear, 2=white, 3=yellow, 4=blood).**

Columns with different superscripts are significantly different: A, B P<0.01; a, b, c P<0.05.
