# Supplementary material for: Behaviour during transportation predicts stress response and lower airway contamination in horses
Source: PLoS One. 2018 Mar 22;13(3):e0194272. doi: 10.1371/journal.pone.0194272 (PMC5863983; doi:10.1371/journal.pone.0194272)
Supplement: S3 Fig — (DOCX) [file pone.0194272.s009.docx]

**S3 Fig**. **Effect of the Time on the distribution of the TW turbidity score (0= transparent, 1= clouded, 2= smoked glassed, 3= opaque).**

Columns with different superscripts are significantly different: A, B P<0.01; a, b, c P<0.05.
